# Ziprasidone Induces Rabbit Atrium Arrhythmogenesis via Modification of Oxidative Stress and Sodium/Calcium Homeostasis

**DOI:** 10.3390/biomedicines10050976

**Published:** 2022-04-23

**Authors:** Buh-Yuan Tai, Ming-Kun Lu, Hsiang-Yu Yang, Chien-Sung Tsai, Chih-Yuan Lin

**Affiliations:** 1 Jianan Psychiatric Center, Ministry of Health and Welfare, Tainan 717, Taiwan; tai1337@hotmail.com (B.-Y.T.); msdrkun@yahoo.com.tw (M.-K.L.); 2 Department of Pharmacy, Chia Nan University of Pharmacy & Science, Tainan 717, Taiwan; 3 Department of Biochemistry, National Defense Medical Center, Taipei 114, Taiwan; linrock@ms26.hinet.net; 4 Division of Cardiovascular Surgery, Department of Surgery, Tri-Service General Hospital, National Defense Medical Center, Taipei 114, Taiwan; sung1500@mail.ndmctsgh.edu.tw; 5 Department and Graduate Institute of Pharmacology, National Defense Medical Center, Taipei 114, Taiwan

**Keywords:** atypical antipsychotics, Ca^2+^ and Na^+^ regulation, atrial fibrillation

## Abstract

Background: Atypical antipsychotics increase the risk of atrial arrhythmias and sudden cardiac death. This study investigated whether ziprasidone, a second-generation antipsychotic, affected intracellular Ca^2+^ and Na^+^ regulation and oxidative stress, providing proarrhythmogenic substrates in atriums. Methods: Electromechanical analyses of rabbit atrial tissues were conducted. Intracellular Ca^2+^ monitoring using Fluo-3, the patch-clamp method for ionic current recordings, and a fluorescence study for the detection of reactive oxygen species and intracellular Na^+^ levels were conducted in enzymatically dissociated atrial myocytes. Results: Ziprasidone-treated atriums showed sustained triggered activities after rapid pacing, which were inhibited by KN-93 and ranolazine. A reduced peak L-type Ca^2+^ channel current and enhanced late Na^+^ current were observed in ziprasidone-treated atrial myocytes, together with an increased cytosolic Na^+^ level. KN-93 suppressed the enhanced late Na^+^ current in ziprasidone-treated atrial myocytes. Atrial myocytes treated with ziprasidone showed reduced Ca^2+^ transient amplitudes and sarcoplasmic reticulum (SR) Ca^2+^ stores, and increased SR Ca^2+^ leakage. Cytosolic and mitochondrial reactive oxygen species production was increased in atrial myocytes treated with ziprasidone. TNF-α and NLRP3 were upregulated in ziprasidone-treated myocytes, and the level of phosphorylated calcium/calmodulin-dependent protein kinase II protein was increased. Conclusions: Our results suggest that ziprasidone increases the occurrence of atrial triggered activity and causes intracellular Ca^2+^ and Na^+^ dysregulation, which may result from enhanced oxidative stress and activation of the TNF-α/NLRP3 inflammasome pathway in ziprasidone-treated myocytes.

## 1. Introduction

Ziprasidone is a second-generation antipsychotic (SGA) used to treat schizophrenia and bipolar disorder [1]. In clinical practice, patients taking antipsychotics have an increased risk of cardiomyopathy, myocarditis, arrhythmias, and even life-threatening cardiac events, with a 1.53-fold increased risk of sudden cardiac death (SCD) [2,3,4]. A multicenter drug-surveillance program for cardiovascular adverse reactions (CARs) during antipsychotic treatment revealed that CARs were the highest during treatment with ziprasidone [5]. Ziprasidone has also been shown to induce a high risk for cardiac arrhythmias [6]. Patients with administered ziprasidone had increased heart rates according to electrocardiogram analysis in an observational study [7]. Among antipsychotic-associated arrhythmias, sinus tachycardia is the most common type, followed by atrial fibrillation (AF) and ventricular extrasystoles [5].

While antipsychotics’ involvement in atrial arrhythmias has not been well investigated, a national database analysis demonstrated that exposure to antipsychotics, especially SGAs, increased the occurrence of AF [8]. While antipsychotic-treatment-associated QT prolongation, which produces an increased rate of SCD, is one of the major concerns in psychiatric patients, AF has been shown to facilitate the induction of ventricular arrhythmias, which, in turn, increases the risk of SCD [9,10]. However, the mechanism for ziprasidone-induced atrial arrhythmias, especially in terms of cardiomyocyte ionic regulation and Ca^2+^ cycling, remains poorly understood, and further investigation is needed [5].

The objective of the present study was to evaluate the atrial electrophysiological characteristics, cardiomyocyte Ca^2+^ and Na^+^ homeostasis, and levels of oxidative stress/inflammasomes under ziprasidone treatment. We hypothesized that ziprasidone caused intracellular Ca^2+^ and Na^+^ dysregulation and oxidative stress, leading to atrial arrhythmogenesis. Our results suggest that ziprasidone impaired homeostasis of intracellular Ca^2+^ and Na^+^ homeostasis, enhanced reactive oxygen species (ROS) production, and activated the tumor necrosis factor alpha/NOD-, LRR-, and pyrin domain-containing protein 3 (TNF-α/NLRP3) inflammasome signaling pathway in atrial myocytes, which provides proarrhythmogenic substrates.

## 2. Methods

### 2.1. Preparation of Atrial Tissues for Electromechanical and Pharmacological Tests

All the animal experiments were reviewed and approved by the Institutional Animal Care and Use Committee of the National Defense Medical Center, Taipei, Taiwan (IACUC-22-061), and were conducted in accordance with the Guide for the Care and Use of Laboratory Animals guidelines of the National Institute of Health.

Male New Zealand white rabbits (weight, 2–3 kg; age, 6–8 months) were used in the present study. The rabbits were kept in stainless steel cages in a controlled environment (20–22 °C; 50–70% humidity) under a 12:12 h light–dark cycle with ad libitum access to standard food and deionized drinking water.

The rabbits were anaesthetized using an intramuscular injection of a mixture of zoletil 50 (10 mg/kg) and xylazine (5 mg/kg), with an overdose of inhaled isoflurane (5% oxygen) in a vaporizer, after which they were killed. The anesthetic dose was confirmed as adequate after the rabbits did not exhibit corneal reflexes and motor responses to pain stimuli induced with a scalpel tip. After heparin (1000 units/kg) was administered intravenously, the hearts were harvested through midline thoracotomy, as described previously [11]. For the LA preparation, the LA was opened by an incision along the mitral valve annulus and extending from the coronary sinus to the septum in normal Tyrode’s solution composed (in mM) of 137 NaCl, 4 KCl, 15 NaHCO_3_, 0.5 NaH_2_PO_4_, 0.5 MgCl_2_, 2.7 CaCl_2_, and 11 dextrose gases, with a mixture of 95% O_2_/5% CO_2_. The dissected LA tissue was pinned with needles onto the bottom of a tissue bath. The other end of the LA tissue was connected to a Grass FT03C force transducer (Grass Instrument Co., Quincy, MA, USA) with silk thread. The epicardial side of the LA preparations faced upward. The preparations were superfused with normal Tyrode’s solution at a constant rate (3 mL/min) and saturated with a 95% O_2_/5% CO_2_ gas mixture. The bath temperature was maintained at 37 °C. Before the electrophysiological assessments, the preparations were allowed to equilibrate for 1 h in the bath.

The transmembrane action potentials were recorded using glass microelectrodes (filled with 3M KCL) connected to a WPI Duo 773 electrometer (World Precision Instruments, Sarasota, FL, USA), as described previously [12]. The signals were digitally recorded using a data-acquisition system (cut-off frequency of 10 kHz), through a low-pass filter (16-bit accuracy), at a rate of 125 kHz. A pulse stimulation of 1 ms duration was produced using a Grass S48 stimulator (Grass Instruments, Norfolk, MA, USA) through a Grass SIU5B stimulus unit (Grass Instruments). The action-potential durations (APDs) in the left atrial preparations were recorded under 2 Hz-pulse stimulation. The action-potential amplitude (APA) was calculated through the difference between the peak potential of depolarization and the resting membrane potential (RMP). Repolarization extents of 20%, 50%, and 90% of the APA were termed APD_20_, APD_50_, and APD_90_, respectively. Atrial preparations were acutely perfused with ziprasidone (100 μg/mL) at a constant rate to assess the treatment responses. The rapid atrial pacing (RAP) protocol (a 20 Hz pacing rate for 1 s) was performed with or without ziprasidone to evaluate the triggered electric activity evoked by ziprasidone treatment. Triggered activity was defined as the occurrence of spontaneous APs in the absence of electrical stimuli. Burst firing was defined as the occurrence of accelerated spontaneous APs. The occurrence rate, sustained frequency, and duration of the triggered activity after RAP were recorded.

### 2.2. Cardiomyocyte Isolation

Atrial myocytes were enzymatically dissociated, as previously described, with some modifications [13]. Briefly, the rabbits were anesthetized by using an intramuscular injection of a mixture of zoletil 50 (10 mg/kg) and xylazine (5 mg/kg), with an overdose of inhaled isoflurane (5% oxygen). The hearts were procured and cannulated to a Langendorff perfusion apparatus through the aorta at 37 °C. The hearts were initially perfused using NT solution (137 mmol/L NaCl, 1.8 mmol/L CaCl_2_, 0.5 mmol/L MgCl_2_, 5.4 mmol/L KCl, 10 mmol/L glucose, and 10 mmol/L 4-(2 Hydroxyethyl) piperazine-1-ethanesulfonic acid (HEPES), pH adjusted to 7.4 with NaOH) for 10 min, and were then digested using a Ca^2+^-free solution (120 mM NaCl, 5.4 mM KCl, 1.2 mM MgSO_4_, 1.2 mM KH_2_PO_4_, 6 mM HEPES, 10 mM glucose, and 10 mM taurine (pH adjusted to 7.4 using NaOH)) containing 300 units/mL of collagenase (Type I; Sigma-Aldrich, St. Louis, MO, USA) and 0.25 units/mL of proteinase (type XIV; Sigma-Aldrich) for 8 to 12 min. After perfusion, the hearts were removed from the cannulas, and the left atriums were excised. The left atriums were cut into small pieces, gently triturated using a plastic transfer pipette in 50 mL of Ca^2+^-free solution and filtered through a nylon mesh until single cardiomyocytes were obtained. The solution for the dissociated cells was then gradually changed to NT solution. The isolated cells were stored in NT solution at 20–22 °C and were allowed to stabilize in the solution for at least 30 min before the experiments. Rod-shaped myocytes with clear striation and without granulation were used within 6–8 h. To test the effects of ziprasidone, the cells were incubated with ziprasidone (100 μg/mL) for 30 min before intracellular Ca^2+^ monitoring, electrophysiological measurements, assessing intracellular Na^+^ levels, and detecting reactive oxygen species (ROS).

### 2.3. Electrophysiological Measurement

#### I_Ca,L_, and I_Na,L_

The ionic currents of the atrial myocytes were measured using whole-cell configuration patch-clamp techniques with an Axopatch 1D amplifier (Axon Instruments, Foster City, CA, USA), as described previously [14]. A small hyperpolarizing voltage step from a holding potential of –50 mV to a potential of –55 mV for 80 ms was given at the beginning of each experiment. The cell capacitance was calculated as the area under the capacitive current divided by the applied voltage step. The series resistance was electronically compensated at approximately 60–80%. The filling solution of microelectrodes for I_Ca,L_ was composed (in mM) of 130 CsCl, 1 MgCl_2_, 5 MgATP, 10 HEPES, 0.1 NaGTP, and 5 Na_2_-phosphocreatine (pH adjusted to 7.2 with CsOH). The filling solution of microelectrodes for I_Na,L_ was composed (in mM) of 130 CsCl, 4 Na_2_ATP, 1 MgCl_2_, 10 EGTA, and 5 HEPES (pH adjusted to 7.3 with NaOH). The I_Ca,L_ was determined as inward currents during voltage-clamp steps from a holding potential of –50 mV to potentials from –40 to +60 mV in steps of 10 mV for 300 ms at a frequency of 0.1 Hz using a perforated patch clamp with amphotericin B as described previously [15]. The I_Ca,L_ was assessed between 5 and 15 min after formation of perforated whole-cell patch in each cardiomyocyte, to avoid ‘run-down’ effects [15,16]. To measure the I_Na,L_, a step/ramp protocol (starting with a potential of –100 mV, then stepping to +20 mV for 100 ms, and afterwards ramping back to –100 mV for 100 ms) was used. The I_Na,L_ was determined as the tetrodotoxin (30 µM TTX)-sensitive current, obtained when the voltage was ramped back to –100 mV as described previously [15].

### 2.4. Intracellular Ca^2+^ Monitoring

Atrial cardiomyocytes were incubated with Ca^2+^ indicator (10 μM Fluo-3 AM) at room temperature for 30 min, and fluorescent imaging was performed as previously described [17,18]. Briefly, fluorescence microscopy was conducted using an inverted laser-scanning confocal microscope (Zeiss LSM 510; Carl Zeiss, Jena, Germany). The fluorescent signals were corrected for variations in dye concentrations by normalizing the fluorescent signal (represented by *F*) against the baseline fluorescence (*F*_0_) to obtain reliable data about transient intracellular Ca^2+^ changes, denoted as (*F − F*_0_)/*F*_0_, and to exclude variations in the fluorescence intensity caused by different volumes of injected dye. Ca^2+^ transients were elicited using field stimulation at 2 Hz. SR Ca^2+^ stores were measured by fast application of 20 mM caffeine following a pulse stimulation train at 2 Hz for 30 s. The SR Ca^2+^ stores were determined from the peak amplitudes of the caffeine-elicited Ca^2+^ transient. The SR Ca^2+^ leak was measured as the tetracaine (1 mM)-induced reduction in intracellular Ca^2+^, as previously described [19]. Briefly, after steady-state Ca^2+^ transients with repeated pulses (2 Hz for 15 s), the superfusate was rapidly changed to a 0 Na^+^/0 Ca^2+^ solution, composed of (in mM) 140 LiCl, 0.5 MgCl_2_, 5.4 KCl, 10 glucose, 10 EGTA, and 10 HEPES (pH adjusted to 7.4, with LiOH)) containing 1 mM tetracaine, which was applied for a minimum of 20 s to produce reduction in intracellular Ca^2+^

### 2.5. Measurement of ROS Production and Cytosolic Na^+^ Levels

Atrial myocytes were incubated with 10 μM CellROX green, 2 μM MitoSOX Red (Life Technologies, Carlsbad city, CA, USA), and 5 µM Asante NaTRIUM Green-2 AM (Teflabs, Austin, TX, USA) in NT solution to assess the cytosolic and mitochondrial ROS production and the cytosolic Na^+^ level, respectively. The measurements were carried out using an inverted laser-scanning confocal microscope (Zeiss LSM 510, Carl Zeiss, Oberkochen, Germany) as previously described [20]. Light with a wavelength of 488 nm for excitation was used, and the emission fluorescence was detected at wavelengths over 505 nm in the XY mode of the confocal microscope setup. During the experiment, atrial myocytes were paced at 2 Hz. The fluorescent signals were analyzed using ImageJ, as described previously [21].

### 2.6. Western Blot Analysis

Western blotting was carried out to assess the levels of cytokines, inflammasome markers, and Ca^2+^ regulatory proteins, including NF-κB (p65), TNF-α, NLRP3, sarcoplasmic/endoplasmic reticulum Ca^2+^ ATPase 2a (SERCA2a), and calcium/calmodulin-dependent protein kinase II (CaMKII). Whole-cell lysates were prepared from left atrial cardiomyocytes. The cell lysis buffer was composed of 100 mM Tris-HCl (pH 8.0), 0.1% sodium dodecyl sulfate, 1% Triton X-100, 150 mM NaCl, and a protease inhibitor cocktail (Roche, Basel, Switzerland). The myocytes protein extracts were separated by conventional gel electrophoresis, and were then transferred to polyvinylidene difluoride membranes (Merck Millipore, Burlington, MA, USA). After blocking with 5% non-fat milk using TBST (50 mM Tris-HCl (pH 8.0), 150 mM NaCl, and 0.05% Tween 20) at room temperature for 1 h, the membranes were incubated with the following primary antibodies: NF-κB (sc-372; Santa Cruz Biotechnology, Dallas, TX, USA), TNF-α (17590-1-AP; Proteintech, Manchester, UK), NLRP3 (ab-214185; Abcam, Cambridge, UK), SERCA2a (sc-376235; Santa Cruz Biotechnology, Dallas, TX, USA), p-SERCA2a (A010-25AP; Badrilla Ltd., Leeds, UK), p-CaMKII (ab32678; Abcam, Cambridge, UK), and GAPDH (60004-1-ig; Proteintech, Manchester, UK). Subsequently, the membranes were incubated with anti-mouse (AP160P; Millipore, MI, USA) or anti-rabbit (AP132P; Millipore, MI, USA) secondary IgG antibodies. Immunoreactive proteins were detected using enhanced chemiluminescence (GE Healthcare, Chicago, IL, USA) and analyzed using the ImageJ software 1.5 (NIH, Bethesda, MD, USA).

### 2.7. Data Analysis

Student’s *t*-tests or Pearson’s chi-square tests using SigmaPlot version 12 (Systat Software, San Jose, CA, USA) were used to exam the differences between treatments. The ‘n’ represents the numbers of total cells from the total hearts (*n* = cells/hearts), and the ‘N’ is the animal number. Statistical significance is denoted as *, **, and *** for *p* < 0.05, *p* < 0.01, and *p <* 0.005, respectively.

## 3. Results

### 3.1. Atrial-Tissue Electrical Activity

**Figure 1:**
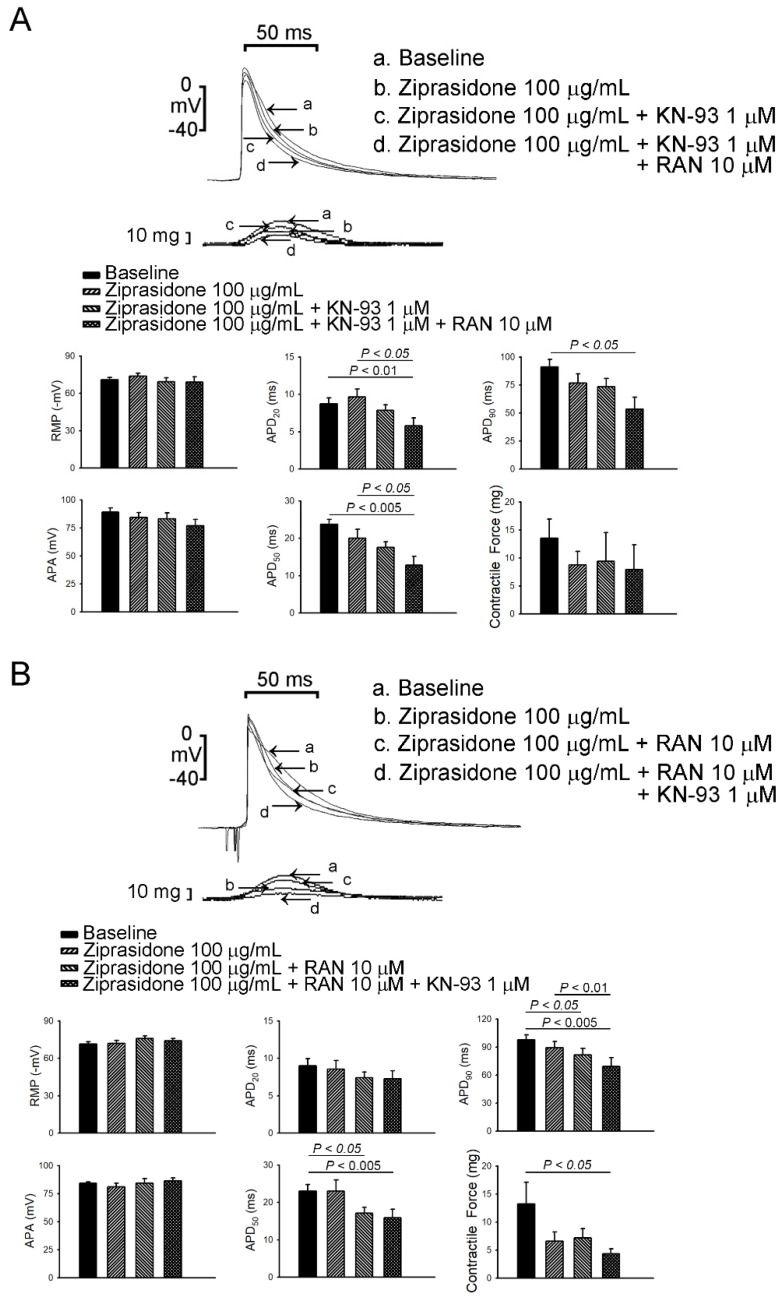
Effects of ziprasidone on action potentials of rabbit atriums. (**A**): (Upper) Representative action potentials and contractile forces from atrial tissues treated with ziprasidone, combined with KN-93 or KN-93 plus ranolazine, and (below) the mean data (N = 7) during electrical stimulation at a rate of 2 Hz. (**B**): (Upper) Representative action potentials and contractile forces from atrial tissues treated with ziprasidone, combined with ranolazine or ranolazine plus KN-93, and (below) the mean data (N = 7) during electrical stimulation at a rate of 2 Hz.

**Figure 2:**
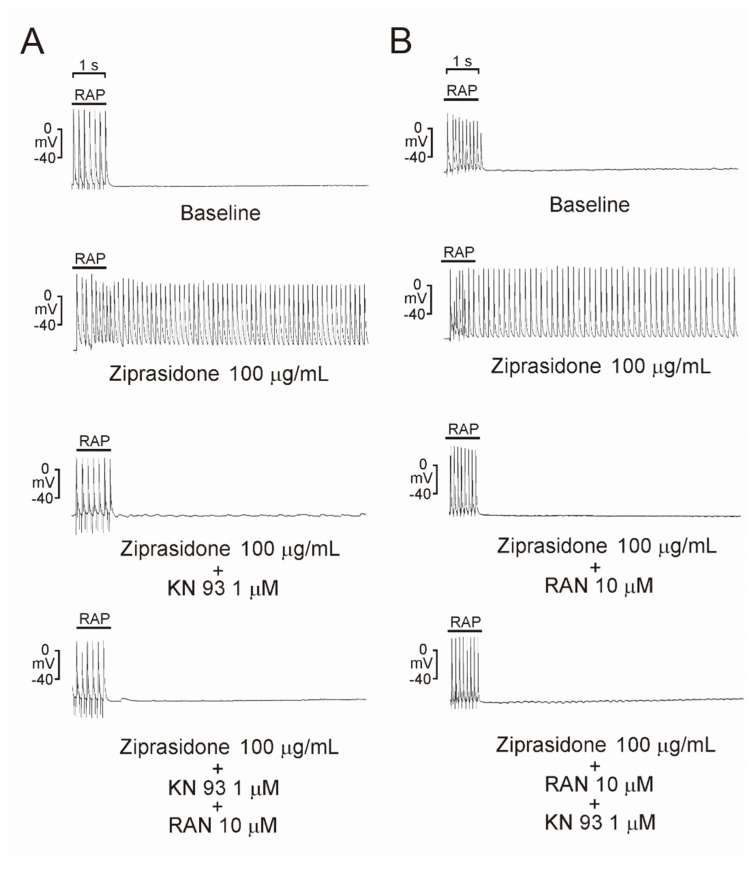
Triggered activity in atrial tissues with ziprasidone. Representative triggered activities in atrial tissues treated with ziprasidone. (**A**): Atrial tissues showed triggered activity with ziprasidone (100% occurrence rate, 7.5 ± 0.4 Hz sustained frequency and 19.8 ± 7.9 s sustained duration, *n* = 7, compared with 0% in atrial tissues without ziprasidone, *n* = 7). KN-93 and KN-93 with ranolazine inhibited the occurrence of triggered activity (0%, *n* = 7). (**B**): Atrial tissues showed triggered activity with ziprasidone (100% occurrence rate, 7.1 ± 0.5 Hz sustained frequency and 22.4 ± 10.4 s sustained duration, *n* = 7 compared with 0% in atrial tissues without ziprasidone, *n* = 7). Ranolazine and ranolazine with KN-93 inhibited the occurrence of triggered activity (0%, *n* = 7).

To investigate whether ziprasidone treatment induced atrial arrhythmia, we first assessed the effect of ziprasidone on the configuration of the action potentials in atriums. Ziprasidone did not affect the RMP, APA, APD_20_, APD_50_, or APD_90_ in the atrial tissues (Figure 1A,B). KN-93 or ranolazine did not show an effect on RMP, APA, APD_20_, APD_50_, or APD_90_ in the ziprasidone-treated atrial tissues (Figure 1A,B). However, ziprasidone-treated atrial tissues showed post-RAP triggered activity, with a 100% occurrence rate, compared to 0% in pre-ziprasidone treated atrial tissues (Figure 2A,B). KN-93, a CaMKII inhibitor, and ranolazine, a late sodium-current inhibitor, inhibited the occurrence of post-RAP triggered activity in ziprasidone-treated atrial tissues (0% occurrence rate) (Figure 2A,B).

### 3.2. I_Ca,L_, I_Na,L_, and Cytosolic Na^+^ Levels

**Figure 3:**
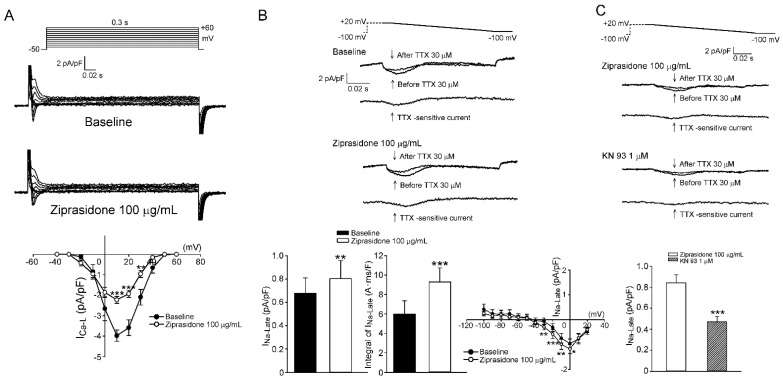
I_Ca,L_ and I_Na,L_. (**A**): (top and middle section) Representative traces of I_Ca,L_ in atrial myocytes with or without ziprasidone and (bottom section) the I–V relationship (*n* = 9/3; ** *p* < 0.01; *** *p <* 0.005). (**B**): (top and middle section) Representative traces of I_Na,L_ (TTX-sensitive current) in atrial myocytes with or without ziprasidone, and (bottom section) the mean data of I_Na,L_ and the integral of I_Na,L_ and the IV relationship (*n* = 9/3; * *p* < 0.05; ** *p* < 0.01). (**C**): (top and middle section) Representative traces of I_Na,L_ in ziprasidone-treated atrial myocytes with or without KN-93, and (bottom section) the mean data (*n* = 13/3; *** *p* < 0.005).

We then tested if ziprasidone affected the myocyte Ca^2+^ influx and late Na^+^ current. The current density of I_Ca,L_ in the atrial myocytes was smaller after ziprasidone treatment (Figure 3A). The I_Na,L_ (tetrodotoxin-sensitive current) in the atrial myocytes was enhanced after ziprasidone treatment (0.68 ± 0.11 pA/pF to 0.79 ± 0.13 pA/pF; ** *p* < 0.01; the integral of I_Na,L_: 5.97 ± 1.37 A*ms/F to 9.29 ± 1.45 A*ms/F; *** *p* < 0.005; Figure 3B). KN-93 reduced the ehnanced I_Na,L_ in ziprasidone-treated myocytes (*** *p* < 0.005*;* Figure 3C). In addition, the intracellular Na^+^ concentration ([Na^+^]_i_) in the atrial cardiomyocytes was higher after ziprasidone treatment (153 ± 6 Δ*F*/*F*_0_ and 172 ± 4 Δ*F*/*F*_0_, respectively; * *p* < 0.05; Figure 5A).

### 3.3. Ca^2+^ Transient Amplitudes, SR Ca^2+^ Stores, and SR Ca^2+^ Leak

**Figure 4:**
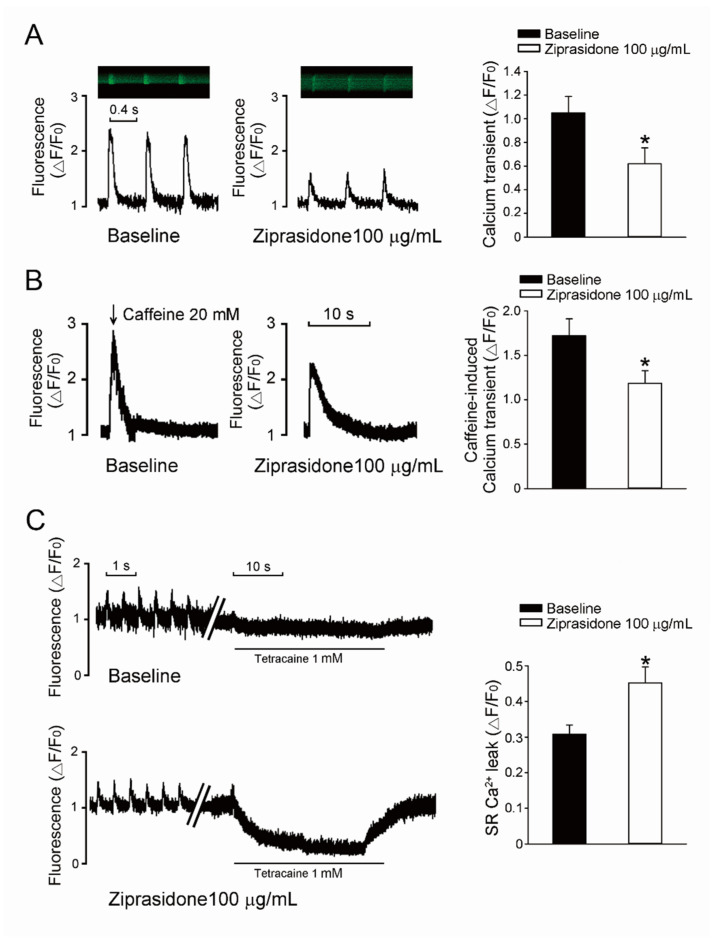
SR Ca^2+^ leak. (**A**): Typical traces of steady-state Ca^2+^ transient in atrial myocytes treated with or without ziprasidone, and mean data (baseline group *n* = 22/3, ziprasidone group *n* = 30/3; * *p* < 0.05). (**B**): A: Typical traces of caffeine-provoked Ca^2+^ transient in atrial myocytes treated with or without ziprasidone, and mean data (baseline group *n* = 22/3, ziprasidone group *n* = 16/3; * *p* < 0.05). (**C**): Typical recording of SR Ca^2+^ leakage determined by fast tetracaine application in atrial myocytes treated with or without ziprasidone, and mean data (baseline group *n* = 13/3, ziprasidone group *n* = 12/3; * *p* < 0.05).

Next, we examined if the intracellular Ca^2+^ regulation machinery was attenuated by ziprasidone. The steady-state Ca^2+^ transient amplitudes in atrial myocytes were smaller after treatment with ziprasidone (* *p* < 0.05, Figure 4A). Ziprasidone-treated myocytes showed decreased SR Ca^2+^ stores, which were assessed based on the Ca^2+^ transient elicited by rapid caffeine application (* *p* < 0.05, Figure 4B) and increased SR Ca^2+^ leakage (* *p <* 0.05, Figure 4C).

### 3.4. Oxidative Stress

**Figure 5:**
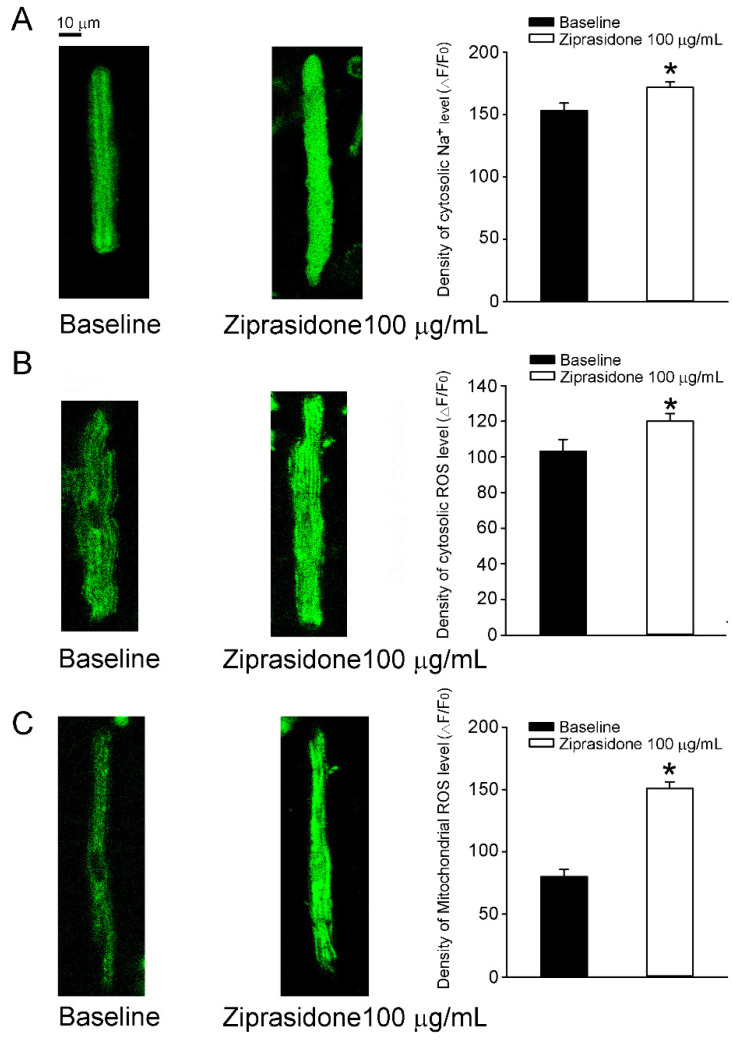
Cytosolic Na^+^ level and oxidative stress in atrial myocytes treated with or without ziprasidone. (**A**): Fluorescent images of cytosolic Na^+^ levels in atrial myocytes treated with or without ziprasidone and mean data (baseline group *n* = 26/3, ziprasidone group *n* = 37/3; * *p* < 0.05). (**B**): Fluorescent images of cytosolic levels of reactive oxygen species (ROS) in atrial myocytes treated with or without ziprasidone and mean data (baseline group *n* = 11/3, ziprasidone group *n* = 29/3; * *p* < 0.05). (**C**). Fluorescent images of mitochondrial ROS levels in atrial myocytes treated with or without ziprasidone and mean data (baseline group *n* = 26/3, ziprasidone group *n* = 32/3; * *p* < 0.05).

The cytosolic ROS levels in atrial cardiomyocytes were increased by 25% after ziprasidone treatment (108 ± 8 Δ*F*/*F*_0_ and 135 ± 6 Δ*F*/*F*_0_, respectively; * *p* < 0.05) (Figure 5B). The mitochondrial ROS levels in atrial cardiomyocytes were enhanced by 87% after ziprasidone treatment (80 ± 6 Δ*F*/*F*_0_ and 150 ± 6 Δ *F*/*F*_0_, respectively; * *p* < 0.05) (Figure 5C).

### 3.5. Levels of Proinflammatory Cytokines, Inflammasome Markers, and Ca^2+^ Regulatory Proteins

**Figure 6:**
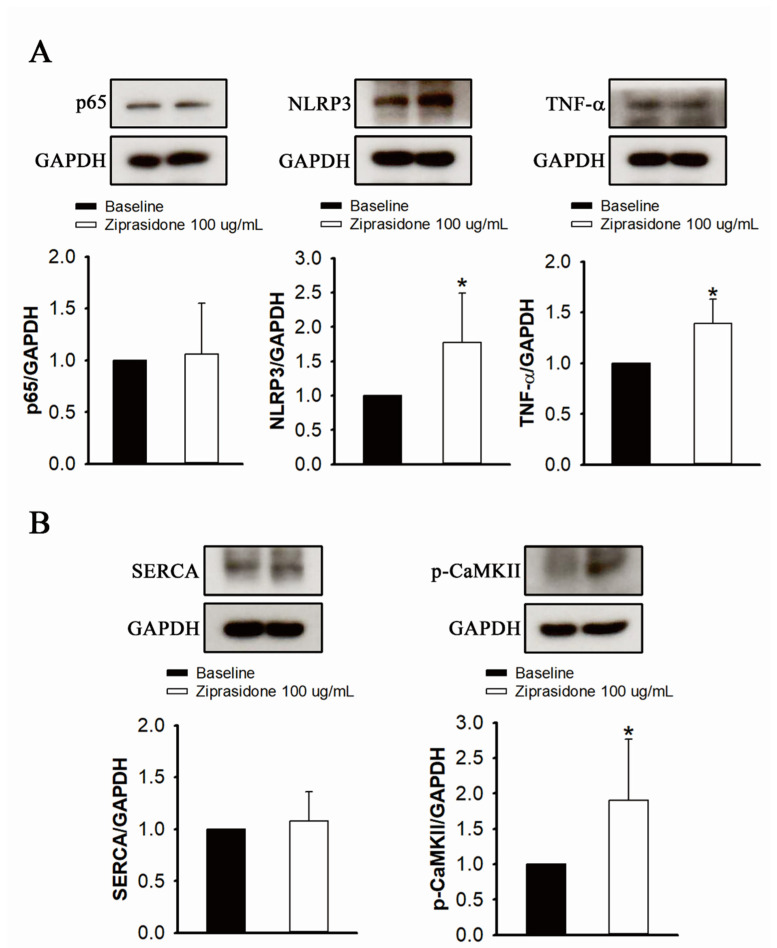
Proinflammatory cytokine and inflammasome markers, and Ca^2+^ regulatory proteins, in control and ziprasidone-treated atrial myocytes. (**A**): Representative immunoblot (upper section) and normalized densitometric p65, NLRP3 and TNF-α protein levels in control and ziprasidone-treated atrial myocytes. (**B**): Representative immunoblot (upper section) and normalized densitometric SERCA2a and phosphorylated CaMKII protein levels in control and ziprasi-done-treated atrial myocytes. GAPDH was used as an internal control (control group *n* = 6 and ziprasidone group *n* = 6; * *p* < 0.05).

While the protein levels of NF-κB (p65) did not change in ziprasidone-treated myocytes, the expression of TNF-α and NLRP3 was upregulated (Figure 6A). Ziprasidone-treated myocytes showed no changes in the protein level of SERCA2a, but an increase in the protein level of phosphorylated CaMKII was observed (Figure 6B).

## 4. Discussion

**Figure 7:**
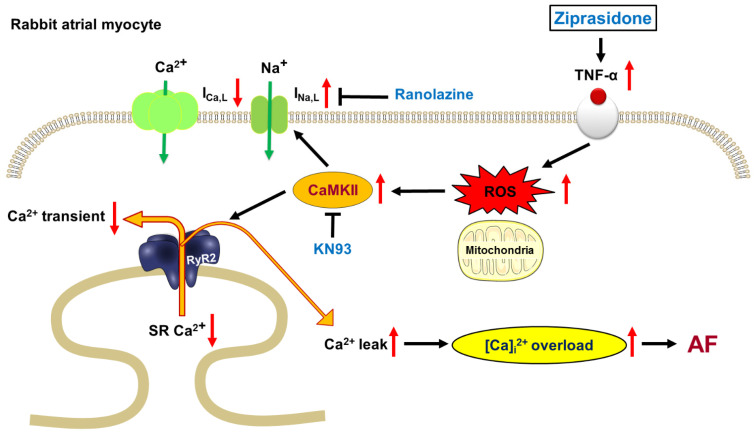
Proposed mechanism for ziprasidone-induced AF. TNF-α were upregulated in ziprasidone-treated myocytes, which induced cytosolic and mitochondrial ROS production. The increased ROS production activated CaMKII, leading to enhanced I_Na,L_ and increased SR Ca^2+^ leak. As a result, intracellular Ca^2+^ was overloaded, promoting AF.

In this study, we used rabbit atrial tissues and isolated cardiomyocytes to assess whether ziprasidone promoted atrial arrhythmias and altered myocyte Ca^2+^ and Na^+^ homeostasis. Our data demonstrate that ziprasidone provoked post-RAP atrial triggered activities, which were suppressed by KN-93 and ranolazine. Ziprasidone reduced the I_Ca,L_ and enhanced the I_Na,L_, as well as increased the intracellular Na^+^ level. Ziprasidone decreased the Ca^2+^ transient and SR Ca^2+^ stores, increased SR Ca^2+^ leakage, and enhanced both cytosolic and mitochondrial ROS production in atrial cardiomyocytes. TNF-α/NLRP3 inflammasome signaling was upregulated in ziprasidone-treated myocytes. These results suggest that ziprasidone impairs the regulation of Ca^2+^, enhances oxidative stress, and activates inflammatory signaling, which leads to the occurrence of arrhythmic events in atriums. These findings help to elucidate the mechanisms underlying atrial arrhythmias associated with ziprasidone treatment (Figure 7).

Ziprasidone-treated atrial myocytes had smaller Ca^2+^ transient amplitudes and lower SR Ca^2+^ stores. We suggest that the smaller SR Ca^2+^ stores may be partly attributed to the lower Ca^2+^-loading effect resulting from the smaller I_Ca,L_, leading to a smaller Ca^2+^ transient and lower SR Ca^2+^ stores [22]. Depleted SR Ca^2+^ stores may also result from an increased probability of ryanodine receptor 2 (RyR2) opening, leading to increased SR Ca^2+^ leakage [23,24]. The CaMKII-mediated hyperphosphorylation of RyR2 enhances diastolic SR Ca^2+^ leakage, which depletes SR Ca^2+^ and increases the cytosolic Ca^2+^ concentration [25,26], which helps to explain the enhanced phosphorylated CaMKII and increased SR Ca^2+^ leakage in the ziprasidone-treated myocytes. Furthermore, CaMKII-mediated SR Ca^2+^ leakage has been shown to promote AF in a mouse model [27]. The overload of cytosolic Ca^2+^ in cardiomyocytes may trigger ectopic electrical activity, such as EADs/DADs, and, consequently, life-threatening arrhythmias [28,29,30]. This creates arrhythmogenic substrates that led to a higher occurrence of triggered activities in the ziprasidone-treated atriums. CaMKII is capable of attenuating intracellular Na^+^ and Ca^2+^ regulation by phosphorylating the related proteins and channels [23,26,31,32,33,34]. The triggered arrhythmia was ameliorated by treatment with the CaMKII inhibitor KN-93 or I_Na,L_ inhibitor ranolazine, which further confirmed the arrhythmogenic role of the upregulated CaMKII and I_Na,L_ induced by ziprasidone.

Enhanced I_Na,L_ contributes substantially to cytosolic Na^+^ levels [35], which may drive NCX to operate in reverse mode to exchange more Ca^2+^ into the cytosol and, as a result, depolarize the membrane potential and increase the probability of ectopic electric activities [36,37]. Increased I_Na,L_ may result from the phosphorylation of the channel Na_v_1.5 by upregulated CaMKII [34,38,39,40]. Triggered activity in ziprasidone-treated atriums was inhibited by an I_Na,L_ inhibitor, ranolazine, which has been shown to reduce intracellular Na^+^-dependent Ca^2+^ overload and ameliorate arrhythmic events [41,42]. Furthermore, increased oxidative stress may also enhance late I_Na,L_, thereby stimulating arrhythmogenesis [43], which may help to explain our findings of enhanced ROS production and increased I_Na,L_ in ziprasidone-treated atrial myocytes.

Oxidative stress has been shown to increase the risk of AF [44,45]. Our data showed that both cytosolic and mitochondrial ROS production increased in ziprasidone-treated myocytes, which may facilitate the occurrence of AF [46]. The SR Ca^2+^ leakage-related overaccumulation of cytosolic Ca^2+^ may result in a mitochondrial overload of Ca^2+^, which, in turn, would exacerbate mitochondrial ROS production. Increased ROS production has been shown to activate CaMKII, which enhances I_Na,L_ and impairs intracellular Ca^2+^ regulation, leading to arrhythmias [47,48]. While there have been reports of ziprasidone exerting an antioxidative-stress effect in neuroblast cells, protecting against neurotoxic-agent-induced apotosis [49,50], treatment with ziprasidone has been reported to induce pathophysiological alterations in the rat heart, which were suggested to be associated with impaired antioxidant capacity due to the ziprasidone [51]. This implies differential pharmacologic effects of ziprasidone on the neurologic and cardiovascular systems.

Our data show that ziprasidone-treated atrial myocytes presented higher levels of TNF-α and NLRP3. Upregulated NLRP3-inflammasome signaling has been shown to promote AF [52,53]. Ziprasidone has also been shown to increase proinflammatory cytokine levels [54]. Ziprasidone exposure induced higher levels of ROS and proinflammatory cytokines, including IL-1, IL-6, TNF-α, and INF-γ, in a macrophage cell line model [54]. Therefore, we suggest that ziprasidone may promote AF via its immunoendocrine effect, which is suggested to be mediated through the TNF-α/NLRP3 pathway.

Finally, some methodological weaknesses in the present study need to be acknowledged. While ranolazine has been well used as an inhibitor of late Na^+^ current, it also inhibits the delayed rectifier potassium current (Ikr) and adrenergic receptors in animal models [55]. However, it should be addressed that the concentration of ranolazine (10 μM) we used in the present study is below the half maximal inhibitory concentration (IC50) of ranolazine for Ikr (11.5 μM) [56]. The widely recognized CaMKII inhibitor KN-93 was shown to reversibly inhibits L-type Ca^2+^ channel in a CaMKII-independent manner [57]. Our data suggest KN-93 inhibits CaMKII, leading to reduction in I_Na,L_ and SR Ca^2+^ leak, which, as a result, producing antiarrhythmic effect in the ziprasidone-treated atriums. I_Ca,L_ were decreased in the ziprasidone-treated myocytes. Therefore, the inhibition effect of KN-93 on L-type Ca^2+^ channel may not be responsible for the primary anti-arrhythmic mechanism in the ziprasidone-treated atriums.

In conclusion, ziprasidone promoted post-rapid-pacing atrial triggered activities. Ziprasidone exposure affected Ca^2+^ and Na^+^ homeostasis in atrial myocytes, leading to a higher occurrence of atrial tachyarrhythmias. Oxidative stress and NLRP3 inflammasomes were enhanced in the ziprasidone-treated myocytes. KN-93 and ranolazine inhibited the atrial arrhythmic events. These findings suggest that ziprasidone causes intracellular Ca^2+^ and Na^+^ dysregulation, which may result from enhanced oxidative stress and an activated TNF-α/NLRP3 inflammasome pathway in ziprasidone-treated myocytes, providing proarrhythmic substrates in atriums.

## Data Availability

The data of the present study are available from the corresponding author upon reasonable request.
